# Early BAL microRNA Signatures Delineate Biological Trajectories Towards CLAD After Lung Transplantation

**DOI:** 10.3390/cells15070611

**Published:** 2026-03-30

**Authors:** Gabriella Gaudioso, Sara Franzi, Riccardo Orlandi, Maria Rosaria De Filippo, Andrea Terrasi, Alessandra Maria Storaci, Nadia Mansour, Barbara Digiuni, Daniele Marchelli, Luca Vittorio Carlo Valenti, Giorgia De Turris, Frederik von Herz, Giulia Garulli, Mario Nosotti, Letizia Corinna Morlacchi, Francesco Blasi, Alessandro Palleschi, Valentina Vaira

**Affiliations:** 1 Division of Pathology, Fondazione IRCCS Ca’ Granda Ospedale Maggiore Policlinico, 20122 Milan, Italy; 2 Division of Thoracic Surgery and Lung Transplantation, Fondazione IRCCS Ca’ Granda Ospedale Maggiore Policlinico, 20122 Milan, Italy; sara.franzi@policlinico.mi.it (S.F.); alessandro.palleschi@unimi.it (A.P.); 3 Laboratory of OMIC Science, Scientific Direction, Fondazione IRCCS Ca’ Granda Ospedale Maggiore Policlinico, 20122 Milan, Italynadia.mansour@unimi.it (N.M.); frederik.vonherz@policlinico.mi.it (F.v.H.); 4 Department of Pathophysiology and Transplantation, University of Milan, 20122 Milan, Italy; daniele.marchelli@unimi.it (D.M.); francesco.blasi@unimi.it (F.B.); 5 Department of Clinical Sciences and Community Health, University of Milan, 20122 Milan, Italy; 6 Precision Medicine Lab, Biological Resource Centre, Transfusion Medicine, Fondazione IRCCS Ca’ Granda Ospedale Maggiore Policlinico, 20122 Milan, Italy; 7 Respiratory and Cystic Fibrosis Unit, Fondazione IRCCS Ca’ Granda Ospedale Maggiore Policlinico, 20122 Milan, Italy

**Keywords:** miRNA, lung transplantation, CLAD, miRNome

## Abstract

**Highlights:**

**What are the main findings?**

- By analyzing microRNA profiles in bronchoalveolar lavage fluid over a long follow-up of 9 years, we identified that early signatures—notably let-7e-5p and miR-30d-3p—are associated with long-term graft failure.
- The study identified distinct miRNA expression patterns that could distinguish between the two main clinical manifestations of chronic graft failure: Bronchiolitis Obliterans Syndrome (BOS) and Restrictive Allograft Syndrome (RAS).

**What are the implications of the main findings?**

- This exploratory pilot study suggests that the biological foundations of Chronic Lung Allograft Dysfunction (CLAD) are established within the first weeks after lung transplantation, long before clinical symptoms appear.
- These molecular changes, linked to pathways like PI3K–mTOR signaling and tissue remodeling, occurred independently of acute rejection episodes, suggesting that early miRNA monitoring could provide a critical window for personalized intervention to improve long-term transplant survival.

**Abstract:**

Chronic lung allograft dysfunction (CLAD) remains the principal limitation to long-term survival after lung transplantation (LT). Early molecular alterations within the graft may precede clinically overt functional decline, but their biological significance remains incompletely defined. In this single-center exploratory pilot study, 16 bilateral lung transplant recipients underwent bronchoalveolar lavage (BAL) sampling at 7 days, 15 days, and 3 months post-transplantation. BAL-derived microRNA (miRNA) profiles were analyzed longitudinally and correlated with long-term clinical outcomes, including CLAD development and phenotypic classification into bronchiolitis obliterans syndrome (BOS) or restrictive allograft syndrome (RAS), over extended follow-up (mean 98 months). Distinct early miRNA signatures were detectable within the first weeks after transplantation and were associated with divergent long-term clinical trajectories. Specific miRNAs, namely let-7e-5p and miR-30d-3p, were associated with subsequent CLAD, whereas differential expression patterns distinguished trajectories toward BOS or RAS. Enrichment analyses highlighted networks related to innate immune activation, hypoxia, tissue remodeling, and PI3K–mTOR signaling. Notably, the occurrence of acute rejection did not differ significantly between patients who developed CLAD and those who remained stable. These findings, although preliminary, suggest that early BAL-derived miRNA profiles may reflect biologically distinct graft states associated with long-term CLAD phenotypes.

## 1. Introduction

microRNAs (miRNAs) are key epigenetic regulators in solid organ transplantation, acting as post-transcriptional modulators that influence both innate and adaptive immune responses. By silencing target messenger RNAs, miRNAs shape T- and B-cell activation, cytokine production, and the generation of donor-specific antibodies [1,2,3]. Their remarkable stability in biofluids and their ability to reflect ongoing molecular events within the graft make them attractive as non-invasive biomarkers of graft injury and long-term outcomes [4].

In lung transplantation (LT), graft dysfunction is increasingly recognized as the culmination of a biological continuum that begins in the donor organ and progresses through early post-transplant inflammatory and immune-mediated insults toward chronic lung allograft dysfunction (CLAD). CLAD, the principal limitation to long-term survival, encompasses two major phenotypes: bronchiolitis obliterans syndrome (BOS) and restrictive allograft syndrome (RAS), both characterized by progressive airway or parenchymal fibrosis [5]. Within this framework, miRNAs have emerged as molecular tracers linking early immune activation, infection, and rejection to subsequent fibroproliferative remodeling of the allograft [6,7].

Pro-fibrotic miRNAs, such as miR-21 and miR-155 [6,7] and the concomitant loss of protective miRNA networks, including the miR-200 family and miR-204 [7], have been implicated in pathways driving epithelial injury, immune dysregulation, and tissue remodeling. These molecular alterations often precede detectable clinical or functional decline, highlighting the potential of miRNAs as early indicators of graft injury. Traditional monitoring tools, including spirometry and transbronchial biopsy, are limited by sensitivity, sampling error, and procedural risk, emphasizing the need for minimally invasive biomarkers. The search for non-invasive biomarkers has shifted toward circulating and exosome microRNAs, which can be detected in blood or bronchoalveolar lavage (BAL) fluid long before a clinical decline in lung function is measurable [6]. Research has identified specific signatures—such as the upregulation of miR-142-5p and miR-155—that can accurately discriminate between stable recipients and those experiencing subclinical rejection [8].

Our previous work [9] has shown that BAL-derived miRNA signatures are associated with acute rejection and infection and can predict one-year outcome after lung transplantation. Building on this evidence, we hypothesized that early BAL miRNA signatures persist over time and remain associated with long-term outcomes, particularly the development of CLAD. In the present study, we therefore aimed to preliminarily investigate whether longitudinal BAL miRNA signatures delineate biological trajectories associated with CLAD during long-term follow-up in a pilot study.

## 2. Materials and Methods

### 2.1. Patients Characteristics

This was a single-center, observational pilot study involving adult lung transplant recipients undergoing routine post-transplant surveillance at the Thoracic Surgery and Lung Transplantation Unit of Fondazione IRCCS Ca’ Granda Ospedale Maggiore Policlinico, Milan. Only adult patients undergoing bilateral lung transplantation from May 2017 to January 2018 were included. Exclusion criteria comprised active infection at the time of BAL sampling and multi-organ transplantation.

The study population reflects the cohort previously described in our earlier report [9]. Briefly, patients were enrolled during scheduled surveillance bronchoscopies and followed longitudinally for clinical outcomes. BAL samples were collected at 7 days (T0), 15 days (T1), and 3 months (T2) after transplantation. BAL was performed according to ISHLT standards [10]. The study was approved by the local Ethics Committee (Protocols IDs 640/2017bis, 2751/2022 and 11901/2025), and all patients provided written informed consent. Clinical variables collected included primary lung disease, age at transplantation, sex, lung allocation score, and cytomegalovirus serology. Donor-related variables comprised age, sex, smoking history, type of donation, Oto score, cytomegalovirus serology, donor–recipient sex mismatch, and the use of static or portable machine perfusion. Surgical variables included intraoperative extracorporeal membrane oxygenation (ECMO), duration of mechanical ventilation, and warm ischemic time.

During the first post-transplant year, the occurrence of primary graft dysfunction (PGD), best forced expiratory volume in one second (FEV_1_), pneumonia, and histological or clinical acute rejection (AR) episodes were recorded. Patients were followed for up to nine years. Chronic lung allograft dysfunction (CLAD) was defined and classified according to ISHLT criteria [5] based on longitudinal spirometry and clinical assessment. Time to CLAD onset was documented, and analyses were performed considering both 5-year and long-term follow-up endpoints.

The primary objective of this exploratory study was to investigate whether early BAL-derived microRNA profiles delineate distinct biological trajectories of the lung allograft over time. Clinical outcomes, including the development of CLAD, time to CLAD onset, and CLAD phenotypes (BOS or RAS), were analyzed as long-term correlates of early molecular signatures. Given the pilot design and limited sample size, the study was conceived as hypothesis-generating.

### 2.2. BAL Collection and miRNA Analysis

BAL was performed according to standard institutional protocols and ISHLT indications. Samples were immediately placed on ice, centrifuged at 2500 rpm at 4 °C for 15 min to remove cellular debris, and the supernatant was stored at −80 °C until RNA purification.

Briefly, total RNA was purified using the RNeasy serum/plasma column-based kit (Qiagen, Venlo, The Netherlands) adding a spike-in exogenous miRNA (cel-miR-39) to monitor the purity and efficiency of miRNA isolation. Then, 90 ng of total RNA per sample were reverse-transcribed and pre-amplified using the Megaplex Primers Pools A and B (Thermo Fisher Scientific, Waltham, MA, USA). miRNA profiles were obtained using the TaqMan Array Human microRNA Card Set v3.0 (Thermo Fisher Scientific) on an ABI PRISM 7900HT instrument (Thermo Fisher Scientific) as previously described [9].

Only miRNAs present in the miRBASE v22 were considered for the analyses. miRNAs whose mean raw data (Ct) less than one standard deviation was above 35 (mean miRNA Ct − SD > 35) were considered undetermined and excluded from the study. Relative quantities of expressed miRNAs were computed from raw Cts using the global mean method. Then, miRNAs RQ were median-normalized, transformed into positive values through a plus 1 addition, and log2-transformed for downstream statistical analyses.

### 2.3. Data Analysis

miRNA data were evaluated through both unsupervised and supervised methodologies. Analyses were performed within the R statistical computing environment (version 4.5.3), as outlined in [9], utilizing the Biobase, perm, nparLD, survival, FactoMineR, NbClust, and ComplexHeatmap libraries. For unsupervised analysis, normalized miRNA levels were imported into RStudio, and heatmaps were generated with the Bioconductor ComplexHeatmap package (release 3.22) using Euclidean distance and the complete linkage method for clustering. Additionally, the *K*-means clustering algorithm was applied to visualize groups of co-regulated miRNAs at distinct time points.

Principal component analysis (PCA) to visualize LT patients’ distribution according to the miRNome at individual times was performed using the prcomp function, plots were generated using the ggplot2 package (version 4.0.2) and the Permanova test was performed using the adonis2 function from the vegan package, using the distance matrix as input.

Differences in miRNA expression according to CLAD phenotypes (BOS and RAS, treated as categorical variables) were performed either separately at individual time points (T0, T1, and T2) or as variations in each miRNA across T0–T2 time points. For the first analysis, a two-sided permutation test was performed for differential expression using the permTS function of the perm R package (version 1.0-0.4) and the exac.ce method, appropriate for small sample size.

Time-dependent variations in miRNA levels during T0, T1, and T2 were examined in relation to clinical features using the Estimated Marginal Means function (emmeans package version 2.0.2). Specifically, a model with an interaction of time and clinical feature, was considered for each miRNA to evaluate if the levels of miRNAs at T0, T1, and T2 varied differently in patients with BOS or RAS compared with patients who had a stable graft function (Stable).

The accuracy of the data was assessed by a permutation-based estimate of the false discovery rate (FDR). Accordingly, all *p* values were corrected for multiple comparisons using the FDR approach unless otherwise stated.

The associations between categorical variables were investigated using the Chi-squared or Fisher exact test (MedCalc software version 12.5.0.0) as appropriate.

The impact of clinical or molecular variables on the incidence of CLAD, BOS or RAS was analyzed by univariate or multivariate analysis using the Cox proportional-hazards regression model (MedCalc software). The miRNA level cutoffs used to sort LT patients into low- and high-expression miRNA groups were generated using receiver operating characteristic (ROC) curves and Youden’s J statistic, as described [11]. Kaplan–Meier survival curves were compared using log-rank and Gehan–Breslow–Wilcoxon tests. When a multivariate model was performed, the enter method (*p* < 0.1) was used.

Lists of target genes were generated using the miRTargetLink v 2.0 Human tool (https://ccb-compute.cs.uni-saarland.de/mirtargetlink2/, accessed on 9 December 2025), which provides a list of experimentally validated miRNA targets, and imported into the STRING database (https://string-db.org, accessed on 9 December 2025) for functional enrichment analysis. Alternatively, miRNA-affected pathways were analyzed using the Diana miRPath v. 4.0 web tool (http://62.217.122.229:3838/app/miRPathv4, accessed on 7 January 2026), using the targets union function. Data are presented as mean  ±  SEM or median  ±  interquartile range, as specified in the legend.

### 2.4. C19MC microRNAs Analysis in Airway Cells Cocultured with BAL-Derived Extracellular Vesicles

Primary, non-transformed and patient-derived airway cells (HBEpC-c) were purchased from PromoCell (C-12640) and cultured in Airway Epithelial Cell Growth Medium Kit (C-21160, PromoCell, Heidelberg, Germany). HBEpC-c cells were then co-cultured for 48h with one aliquot of BAL-EVs obtained from stable (n = 10) or BOS (n = 8) patients as previously described [11]. Total RNA was then purified using the MasterPure RNA purification Kit (Epicentre, Illumina, San Diego, CA, USA) and the expression of miR-512-3p, miR-517c, miR-520d and miR-523-3p was analyzed by qPCR using specific TaqMan probes (Thermo Fischer Scientifics). The mammary U6 small RNA was used as an internal reference and microRNAs relative quantity (RQ) was calculated using the 2^−DCt^ formula. RQ were then median-normalized and log2-transformed.

## 3. Results

### 3.1. Clinical Population

Sixteen lung transplant recipients transplanted between May 2017 and January 2018 were included in the study. Baseline demographic and clinical characteristics are summarized in Table 1. The cohort comprised 10 males and 6 females, with a median age of 37 years (range 18–64). The primary indication for transplantation was cystic fibrosis (CF) in 12 patients and interstitial lung disease (ILD) in 4 patients. Pre-transplant systemic steroid therapy was ongoing in seven recipients. The mean Lung Allocation Score (LAS) was 41.6 (range 31.0–68.6).

Donors were predominantly donation after brain death (DBD, n = 12), whereas four grafts were obtained from donation after circulatory death (DCD) donors [12]. Ex vivo lung perfusion (EVLP) was performed in six cases. Grade 2–3 primary graft dysfunction (PGD) at 72 h occurred in six patients.

Acute rejection (AR) was documented in eight recipients during follow-up, including six cases within the first post-transplant year. Antibody-mediated rejection occurred in four patients. Pneumonia was diagnosed in seven recipients, mainly during the first year.

Patients underwent long-term follow-up (mean 98 months, range 19–104). Chronic lung allograft dysfunction (CLAD) developed in 10 patients: 7 were classified as bronchiolitis obliterans syndrome (BOS) and 3 as restrictive allograft syndrome (RAS) according to the ISHLT criteria [5]. The median time to CLAD diagnosis was 26 months. The cumulative incidence of CLAD was 44% at 5 years and 62.5% during extended follow-up.

Two patients with CLAD required re-transplantation due to advanced graft dysfunction, and four died during follow-up. Mean survival was shorter in patients who developed CLAD (72 months) compared with stable recipients (96 months). Overall survival rate at last follow-up was 75%.

The incidence of acute rejection was not significantly different between patients who developed CLAD and those who remained stable (6 vs. 2 cases; Fisher’s exact test, *p* = 0.6). Recipients who developed CLAD exhibited lower best FEV_1_ within the first post-transplant year and were more likely to have FEV_1_ values below 80%. BAL samples at T2 were unavailable for three patients due to hemolysis (Supplementary Table S1).

### 3.2. The BAL-miRNome After LT

**Figure 1:**
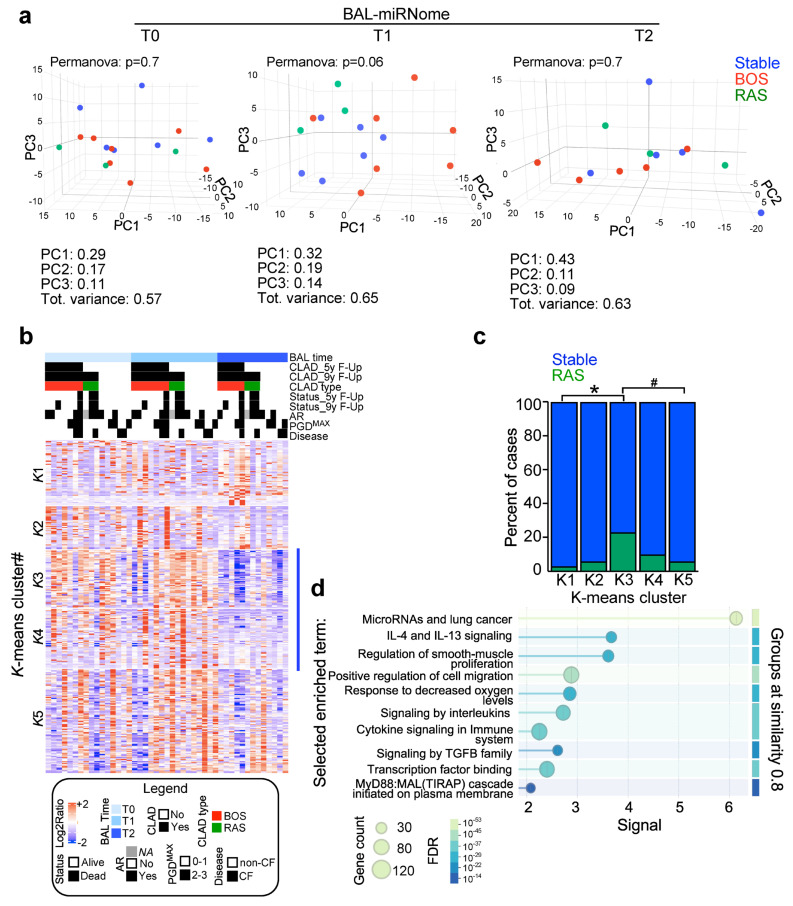
BAL-miRNome after lung transplantation. (**a**) Principal component analysis was performed, with global microRNAs expression evaluated after 7 days (T0), 15 days (T1) or three months (T2) from LT. The first three principal components (PCs) and the associated variance are shown together with Permanova test-associated *p* value. (**b**) Supervised clustering of miRNA expression (n = 317) in LT patients sorted for BAL time and CLAD development during follow-up. Red and blue indicate high and low miRNA expression, respectively. To identify groups of miRNAs with similar behavior, *k*-means clustering analysis was performed, yielding 5 miRNA clusters. (**c**) microRNAs in *k*-means clusters 3 and 4 (see Supplementary Table S2 for details) distinguish LT patients who developed RAS during the 9 yr follow-up. *, *p* adj = 0.015; #, *p* adj = 0.038 by Chi-square test. (**d**), Target prediction of *k*-3 and *k*-4 miRNAs was performed with the miRTargetLink 2.0 Human tool, and gene lists were annotated using String database. Significant terms are presented as bubble plot (see also Supplementary Table S3 for details). AR, acute rejection; CF, cystic fibrosis; PGD, primary graft dysfunction.

Using an unsupervised approach, we analyzed whether the BAL miRNome measures after 7 days (T0), 15 days (T1), or 3 months (T2) from transplantation could discriminate among LT patients according to their outcome. Principal component analysis shows that particularly T1- and marginally T2-miRNome differentiates stable patients from BOS and RAS cases (Figure 1a). By applying a K-means clustering analysis to global microRNA expression (Figure 1b), we identified two miRNA clusters (K3 and K4) with a general low expression in all samples, which was significantly associated with the RAS outcome category (Figure 1c), but not with BOS. Then, the K3 and K4-miRNAs targets were retrieved (Supplementary Table S2) and analyzed for pathways annotation (Supplementary Table S3). Interestingly, interleukin and cytokine signaling, the MyD88 cascade, and regulation of smooth muscle cell proliferation were significantly enriched (Figure 1d), indicating a peculiar lung microenvironment associated with RAS development.

### 3.3. miRNAs Let7e-5p, miR-885-5p and miR-30d-3p Are Concordantly Deregulated in BOS and RAS Compared with Stable Patients

**Figure 2:**
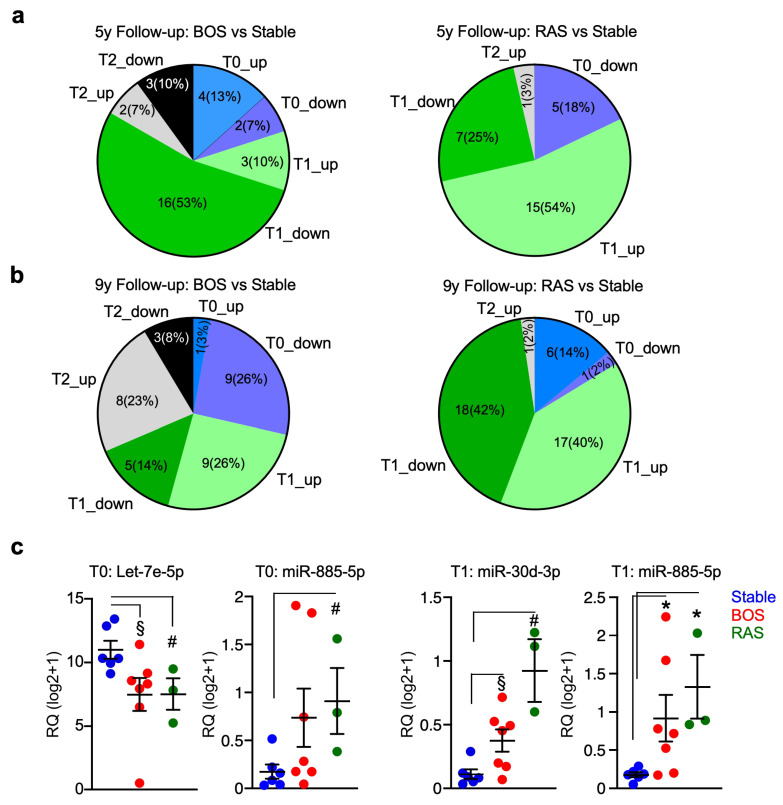
The BAL-miRNAs let-7e-5p, miR-30d-3p and miR-885-5p are concordantly deregulated in LT patients who developed BOS or RAS. (**a**,**b**) Differential miRNAs expression was performed at each time point separately for LT patients who developed BOS and RAS compared with patients who remained stable considering the two CLAD-free survival endpoints, which were 5 (**a**) and 9 (**b**) years. The pie charts depict the percentage of deregulated miRNAs for each comparison, and the number of differentially expressed miRNAs is also shown. (**c**) The expression of the miRNA let-7e-5p and miR-885-5p from BAL sampled at T0 and of the miRNAs miR-30d-3p and miR-885-5p from BAL sampled at T1 was significantly different in LT patients who developed RAS or BOS compared with stable patients. Each dot is a patient; bars, mean ± SEM. *, *p* = 0.01; §, *p* = 0.02; #, *p* = 0.04 by two-sided permutation test.

Given the different global expression of miRNAs in patients who developed BOS or RAS type of chronic allograft dysfunction, we performed supervised analyses for differentially modulated miRNAs at individual time points separately for BOS and RAS cases compared with stable patients. The percentage of miRNAs significantly deregulated at each time differed in the two CLAD types, although the overall number was similar (Supplementary Table S4 and Figure 2a); indeed, BOS showed no specific prevalence of miRNA deregulation at any time point, whereas RAS showed a predominant trend in miRNA deregulation at T1. This behavior was consistent considering both the 5- and the 9-year follow-up periods (Figure 2a,b).

We then searched for commonly deregulated miRNAs, to identify potential key signaling of poor graft outcome. We found that Let-7e-5p was significantly downmodulated at T0 in patients who developed BOS or RAS compared with stable cases; however, miR-885-5p was upmodulated both at T0 and T1 and miR-30d-3p was upregulated at T1 in cases who developed BOS or RAS. Interestingly all those miRNAs are crucial regulators of lung homeostasis, with Let-7e known to prevent excessive tissue remodeling and fibro-inflammation in chronic lung diseases such as chronic obstructive pulmonary disease (COPD) and emphysema [13,14]. Also, miR-30d-3p and miR-885-5p are key mediators of the pulmonary inflammatory response and alveolar cell stress, where their altered levels in systemic circulation reflect the severity of COPD and the progression of idiopathic pulmonary fibrosis [15,16].

**Figure 3:**
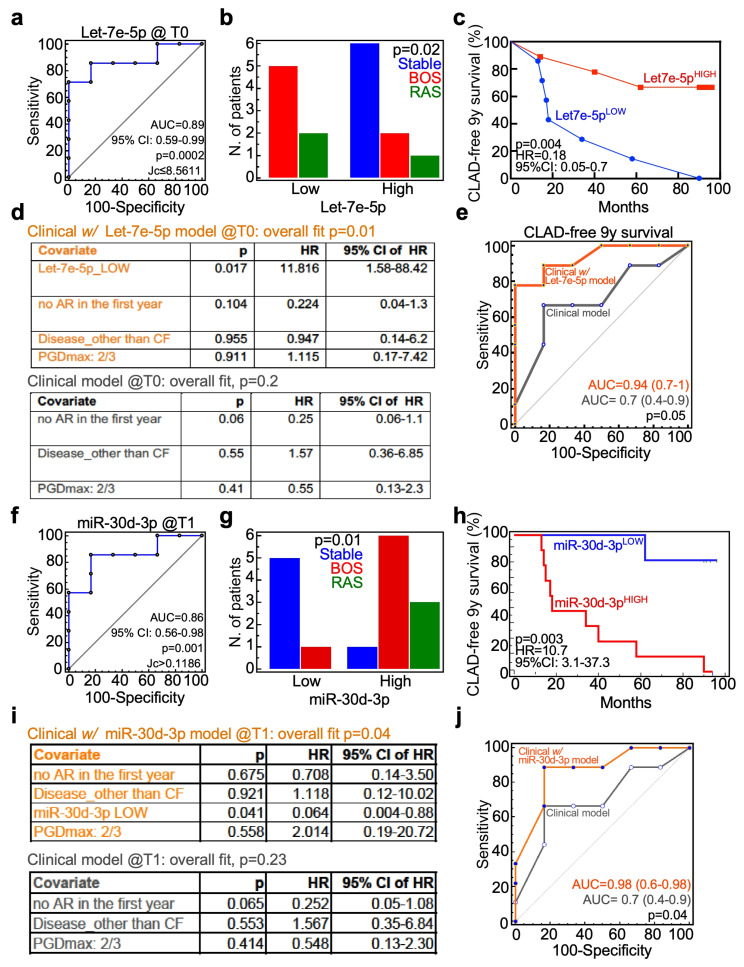
The BAL-microRNA let-7e-5p and miR-30d-3p are novel early biomarkers of CLAD after transplantation. (**a**) An ROC analysis was performed to test the accuracy of let-7e-5p from BAL collected at T0 in classifying LT patients according to the development of CLAD within 9 years of LT. The cut-off (Jc) for miRNA expression was calculated using Youden’s J statistic. (**b**,**c**) The ROC-generated cutoff for let-7e-5p was used to sort LT patients into low- or high-expression groups and to test the ability to classify LT patients into the correct outcome group ((**b**); *p* value is from Fisher’s exact test), or to estimate the CLAD-free survival probability within 9 yr of transplantation follow-up time (*p* value is from the log-rank test). (**d**,**e**) A multivariate Cox regression model was performed with clinical variables and let-7e-5p (treated as a categorical variable; top panel) or clinical variables alone (lower panel). The predicted survival probabilities obtained from each model were then used to determine which model better discriminates between LT patient outcome at 9 yr using ROC analysis and DeLong statistics. (**f**,**g**) As for the let-7e-5p, ROC was used to identify the miR-30d-3p expression value in BAL collected at T1 that better distinguished LT patients with a stable outcome from patients who developed CLAD (**f**), and to sort patients (**g**). The miR-30d-3p expression category was then used to plot CLAD-free survival curves (**h**) and to build a multivariate Cox regression model (**i**). Similarly to let-7e-5p, miR-30d-3p evaluation at T1 significantly improved the identification of CLAD occurrence in LT patients (**j**). AUC, area under the curve; 95% CI, 95% confidence interval; Jc, Youden’s statistics associated criterion; HR, hazard ratio.

Therefore, we investigated whether those miRNAs were indeed associated with LT patients’ outcome. Let-7e-5p expression measured at T0 was able to classify LT patients according to the outcome category, assessed 9 years after surgery (Figure 3a); according to the ROC-generated Youden criterion (Jc), all patients that remained stable during the follow-up (n = 6; Table 1) had a high let-7e-5p expression. On the other hand, five out of seven patients who developed BOS and two out of three patients who developed RAS had a low let-7e-5p expression (Figure 3b). Most importantly, LT patients’ categorization according to let-7e-5p was prognostic (Figure 3c). We therefore tested, in a multivariate model, whether the molecular variable was an independent factor associated with chronic lung allograft dysfunction. Indeed, let-7e-5p was the only variable significantly retained in the model (Figure 3d). Further, the prediction model implemented with let-7e-5p expression class had a higher accuracy in identifying LT patients with higher risk of CLAD than the one with only clinical variables (AUC of 0.94 compared with 0.7; Figure 3e). Similarly, we tested if miR-30d-3p expression measured at T1 was a marker of chronic dysfunction. By ROC analysis, miR-30d-3p was able to classify LT patients according to their 9-year outcome (Figure 3f) and to provide a cut-off (Jc) to sort LT patients into high- or low-expressor groups. In this case, five out of six cases with a stable allograft fell in the low-miR-30d-3p category, together with only one patient who developed BOS (Figure 3g). miR-30d-3p expression categorization was also prognostic, with LT cases with high miRNA expression having a significantly shorter CLAD-free survival (Figure 3h). Furthermore, miR-30d-3p expression category was an independent variable associated with CLAD risk (Figure 3i) and significantly improved the model’s ability to sort LT patients according to the outcome after 9 years from surgery (AUC of 0.98; Figure 3j).

We also tested the let-7e-5p (Supplementary Figure S1a–c) and the miR-30d-3p (Supplementary Figure S1d–f) regarding their ability to identify LT patients at risk of developing BOS or RAS considering the 5-year follow-up period. Both miRNAs retained their prognostic significance (Supplementary Figure S1a,d) but, surprisingly, performed worse as classifiers (Supplementary Figure S1b,c,e,f).

### 3.4. miRNA Variation over T0–T2 Time Is Different Among Patients Who Develop BOS or RAS

**Figure 4:**
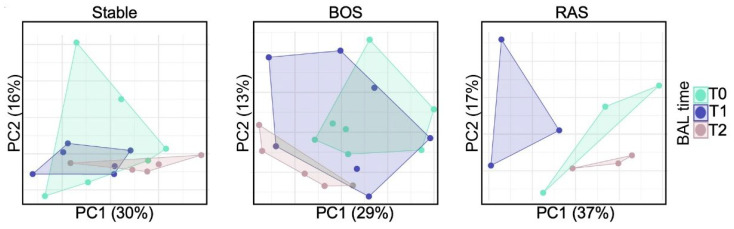
BAL-miRNAs dynamics are different in LT patients according to their outcome. Principal component analysis was performed to understand if the BAL-miRNome measured at the different timepoints diverged among LT patients who remained stable and those who developed BOS or RAS during the 9 yr follow-up. In patients who remained stable, the miRNAs measured at T0, T1 and T2 formed overlapping clouds, while in BOS and RAS cases, miRNAs measured in BAL from different time points clustered separately. PC, principal component.

We then analyzed disease-associated variation in miRNAs over the T0–T2 interval to obtain preliminary insights of miRNA dynamics in the lung allograft environment after transplantation. Overall, miRNAs dynamics differed in patients who did not develop chronic dysfunction compared with patients who developed BOS or RAS (Figure 4). Principal Component Analysis showed that, in stable patients, the miRNome at T0, T1 and T2 formed overlapping clouds, while in patients who developed BOS or RAS miRNA profiles at T0, T1 and T2 were clustered separately (Figure 4). At the supervised level, LT patients with a BOS or RAS outcome showed distinct miRNAs dynamics when compared with stable patients (Supplementary Tables S5 and S6). This analysis suggested that the two different forms of CLAD are characterized by peculiar microenvironments from an early period after LT. We therefore explored miRNA dynamics separately, focusing on miRNAs that were modulated over time, and which were considered continuous variables in LT patients’ BOS- or RAS-free survival (Supplementary Table S7).

### 3.5. C19MC miRNAs and miR-19b-1-5p Expression in the Allograft Environment Is Associated with BOS Development

**Figure 5:**
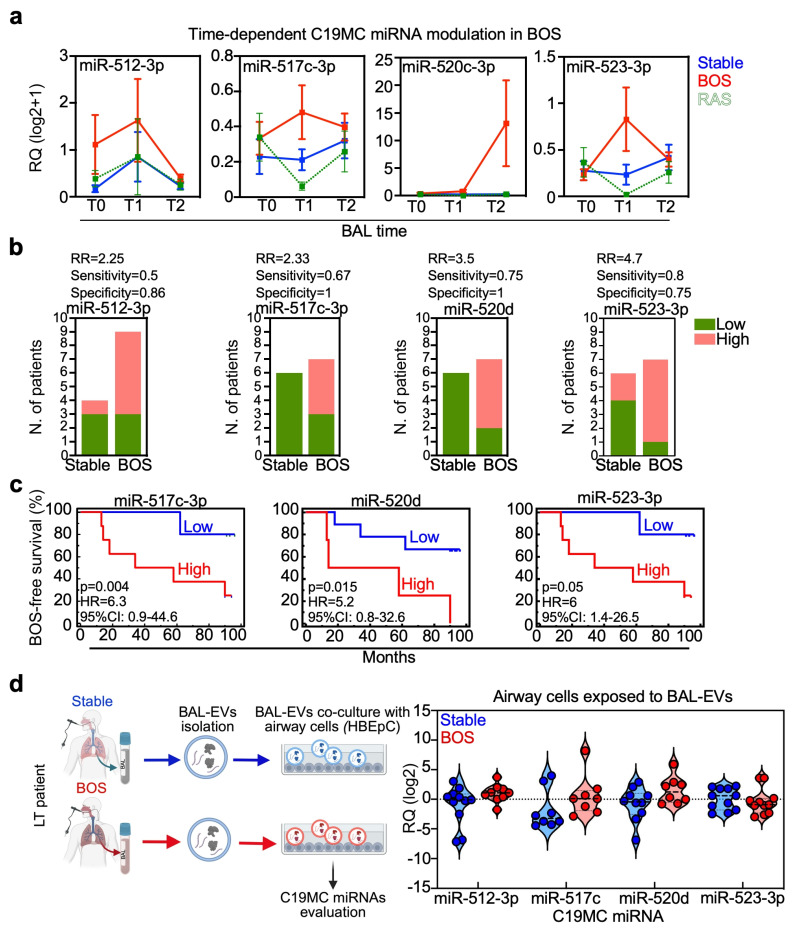
C19MC miRNAs are modulated in BAL from LT patients with higher risk of BOS onset. (**a**) The dynamics of the indicated miRNAs, belonging to the chromosome 19 miRNA cluster, were analyzed in LT patients belonging to the different outcome categories (stable, BOS and RAS). C19MC were significantly modulated in BOS cases (see also Supplementary Table S5 for details). (**b**) C19MC miRNAs expression was analyzed in LT patients belonging to the stable and BOS outcome categories (Table 1) using ROC, and the identified cut-offs (Jc, see Supplementary Figure S2b) were then applied to categorize patients into low- and high-expressor groups. The Relative Risk (RR), sensitivity and specificity was computed for each indicated C19MC miRNA. (**c**) LT patients with high miR-517c-3p, miR-520d, or miR-523-3p have shorter BOS-free survival rates. *p* values are from Log-Rank test; HR, hazard ratios; 95% CI, 95% Confidence Interval. (**d**) The expression of the C19MC miRNAs was assessed in human airway cells (HBEpC) co-cultured for 48 h with BAL-EV from patients with a stable lung function (Stable) or with a diagnosis of BOS. The schematic on the left shows the experimental procedure while the violin plots on the right side show the indicated miRNA expression in the exposed HBEpC cultures. The expression of mir-512-3p, miR-517c and miR-520d is increased in BOS-exposed cells. Each dot is a sample.

LT patients who developed BOS during the 9-year interval had specific increase at T1 of miR-19b-1-5p, miR-512-3p, miR-517c-3p, miR-523-3p, miR-1208 and decrease in miR-451a; at T2, miR-19b-1-5p, miR-190b, miR-374b-3p, miR-380-5p and miR-520c-3p (Figure 5a, Supplementary Figure S2a and Table S5). Interestingly, many of these BOS-associated miRNAs belonged to the primate-restricted and maternally imprinted C19MC cluster on chromosome 19 (Figure 5a, Supplementary Tables S5 and S7), and they are crucial factors in regulating innate immune responses in chronic lung diseases such as asthma and COPD and tissue fibrosis [16].

For all those differentially modulated miRNAs, we tested their potential association with BOS-free survival. To this end, ROC analysis was performed to identify cut-offs and assess if high or low miRNA levels impacted BOS-free survival, always considering both the 5- and the 9-year follow-up periods (Supplementary Figure S2b–e). In general, high C19MC miRNAs expression was associated with BOS development, with a relative risk range of 2.25–4.7 (Figure 5b). Furthermore, high expression of those miRNAs was correlated with shorter BOS-free survival (Figure 5c), but was not independently associated with risk of BOS development in Cox models. Lastly, in our co-culture system of airway cells and BAL-derived extracellular vesicles (BAL-EV) [11], we evaluated if members of the C19MC cluster were upregulated in airway cells after their exposure to BOS-derived BAL-EV compared to EV derived from stable patients. This experiment showed that C19MC miRNA is increased in airway cells compared to BOS-derived BAL-EV (Figure 5d).

Regarding the other BOS-related miRNAs, only miR-19b-1-5p, a member of the miR-17/92 cluster, was both a prognostic and an independent biomarker associated with BOS risk development, showing better performance when the 9-year follow-up time was studied (Supplementary Figure S3a–c).

The remaining miRNAs, miR-1208, miR-1247-5p, miR-451a, were good classifiers but no one was associated with BOS onset (Supplementary Figure S2c–e). On the contrary, miR-328-3p, 374b-3p and miR-380-5p, which were modulated at T2, did not sort LT patients into groups with different BOS development risk levels (Supplementary Figure S2f,g).

Overall, our data show that members of two important miRNA clusters, the C19MC and miR-17/92, are associated with BOS development and they could influence LT patients’ prognosis.

### 3.6. miRNAs Enriched in the Allograft Environment of RAS-Developing Patients Are Involved in mTOR Signaling, Inflammation and Tissue Remodeling

**Figure 6:**
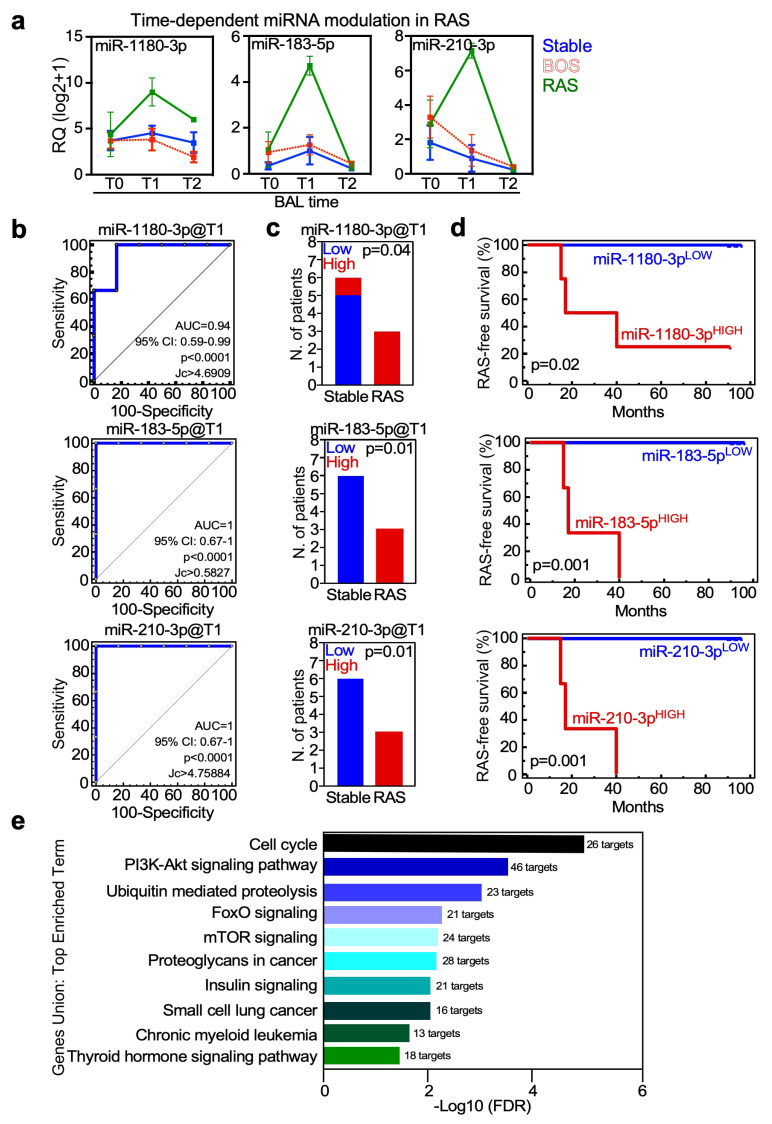
miRNAs significantly deregulated in RAS-developing LT patients. (**a**) Dynamic increase in miR-1180-3p, miR-183-5p and miR-210-3p at T1 characterizes LT patients with RAS outcome (see also Supplementary Table S6 for details). (**b**,**c**) ROC analysis evaluated the diagnostic accuracy of miR-1180-3p, miR-183-5p, and miR-210-3p from BAL fluid at T1 in differentiating stable from RAS-developing LT patients (**b**). The resulting cut-offs (Jc) were used to classify patients as low or high expressors (**c**). High expression of miR-183-5p and miR-210-3p is a unique characteristic of LT patients who developed RAS during the 9y follow-up. (**d**) LT patients with high miR-1180-3p, miR-183-5p or miR-210-3p have a significantly shorter RAS-free survival time than low-expressing patients. (**e**) The targets of miR-1180-3p, miR-30d-3p, miR-183-5p and miR-210-3p, all upregulated at T1 in LT patients who developed RAS, were analyzed in DIANA-miRPath v4.0 using the Genes Union function. The top enriched terms are shown, along with the number of target genes involved in each signaling.

Patients who developed RAS during the follow-up showed increased expression over time of miR-31-3p, miR-183-5p, miR-210-3p, miR-22-3p, miR-449a and miR-1180-3p compared with stable patients, and had lower initial miR-34a-5p levels (Figure 6a, Supplementary Figure S4 and Table S6).

In particular, miR-1180-3p, miR-183-3p and miR-210-3p, were highly associated with RAS development (Figure 6b) and precisely categorized LT patients in the stable or RAS group (Figure 6c). High expression of the three miRNAs was also correlated with shorter RAS-free survival time (Figure 6d and Supplementary Table S7).

We than searched for RAS-related increased miRNAs targets and related signaling. Enriched terms were related to inflammatory and immune-related signaling, such as FoxO and chronic myeloid leukemia, to tissue remodeling, such as proteoglycans in cancer, and to the PI3K-mTOR signaling, a pathway that is the target of treatment currently used for CLAD, such as Everolimus or Sirolimus (Figure 6e). Finally, among enriched terms, we found thyroid hormone signaling. Thyroid hormone supplementation is used to precondition the lung allograft to improve organ viability and procurement [17].

## 4. Discussion

In this exploratory pilot study, we found that early BAL-derived microRNA signatures are associated with distinct long-term clinical trajectories of the lung allograft. Rather than functioning solely as isolated biomarkers, these early molecular profiles may reflect broader network-level biological states established shortly after transplantation, or even during transplantation itself, which are consistent with subsequent development of CLAD.

Differential microRNA patterns were already detectable within the first weeks after transplantation, well before clinically evident functional decline. These early signatures were associated not only with the later occurrence of CLAD but also with its phenotypic heterogeneity, distinguishing trajectories toward BOS and RAS. Although causal relationships cannot be inferred, this early divergence supports the hypothesis that BOS and RAS may represent the clinical expression of distinct biological programs within the graft rather than simply late manifestations of a uniform rejection process [18].

Indeed, the let-7e-5p and miR-30d-3p miRNAs may serve, as confirmed by larger studies, as early indicators for CLAD. Elevated levels of C19MC miRNAs or miR19b-1-5p from the miR-17/92 cluster are linked to BOS development, while the increase in inflammatory and remodeling miRNAs—miR-183-3p, miR-210-3p, and miR-1180-3p—are associated with RAS dysfunction. This observation is particularly relevant considering the clinical heterogeneity of the paths to chronic rejection [18], and suggest that miRNA dysregulation reflects early molecular events in the allograft that are not captured by conventional clinical parameters.

microRNAs are uniquely suited to capture such complex biological states, as they regulate entire signaling networks rather than single pathways. Several of the identified miRNAs have established roles in lung development, inflammation, and tissue remodeling.

The miR-17/92 cluster regulates lung development [19,20], while the Let-7 family exerts tumor-suppressive and homeostatic functions in pulmonary cells [21,22,23], and its dysregulation has been implicated in chronic lung diseases such as COPD and emphysema [14,16]. MiR-30d modulates inflammasome activity and IL-1β secretion, processes relevant to early graft injury. C19MC miRNAs act as regulators of Toll-like receptor (TLR) signaling and innate immune activation [24], pathways central to chronic inflammatory responses [25].

In contrast, RAS-associated miRNAs—including miR-183-3p, miR-210-3p, and miR-1180-3p—have been linked to fibrotic remodeling, Th17 differentiation, and hypoxia-driven responses. MiR-183-3p has been described as a “fibromiR,” influencing T-cell proliferation and IFNγ production [26], besides participating in IL-6/STAT3-dependent pathogenic Th17 responses [27]. MiR-210, the so-called “hypoxamiR,” is a direct target of HIF1α and contributes to pathological remodeling and fibroblast proliferation in pulmonary hypertension and idiopathic pulmonary fibrosis [28,29]. Collectively, these data suggest that early microRNA signatures may integrate signals related to immune activation, hypoxia, and intrinsic graft remodeling processes.

The enrichment of PI3K–Akt and mTOR signaling among RAS-associated targets is particularly relevant, as these pathways are therapeutically targeted in CLAD [30,31]. mTOR activation has been associated with immune cell survival and pro-inflammatory lymphocyte differentiation but also plays a central role in cellular metabolism and proliferation within structural lung cells [11,32]. Similarly, enrichment of thyroid hormone signaling—implicated in cytoprotection against ischemia–reperfusion injury [17] and in the modulation of TGF-driven fibrosis and epithelial-to-mesenchymal transition [33]—supports the possibility that early graft vulnerability may involve metabolic and stress-response pathways beyond alloimmune injury. A recent paper [34] has highlighted that end-stage CLAD shares immunological and fibrotic mechanisms with chronic lung diseases that do not involve lung transplantation. Further, they found a pathogenic epithelial cell type characterized by the expression of cytokeratin 17 and absence of cytokeratin 5 common to CLAD and other fibrotic lung diseases. The authors also suggested that existing anti-fibrotic therapies might also be implemented for LT patients, in addition to immunosuppression treatment. In this context, fibrosis-associated BAL-miRNAs, if confirmed by larger studies, might be used as an early biomarker for the identification of LT patients that could benefit from those therapies.

Importantly, in our cohort, the occurrence of acute rejection did not differ significantly between patients who developed CLAD and those who remained stable. While immune-mediated injury undoubtedly contributes to chronic dysfunction, this observation suggests that clinically overt rejection alone may not fully account for the divergent long-term trajectories observed. Our findings are consistent with the hypothesis that CLAD may emerge from early-established biological states of the transplanted lung, shaped by a complex interplay of donor-related factors, ischemia–reperfusion injury, epithelial resilience, innate immune activation, and subsequent adaptive responses [1,2,3].

Most prior biomarker studies in CLAD have focused on circulating microRNAs measured in serum or plasma [35]. While minimally invasive, circulating profiles may predominantly reflect systemic immune activation. In contrast, BAL fluid samples the local allograft microenvironment [8], providing compartment-specific insight into lung-centered molecular processes. Our data suggest that BAL-derived microRNA signatures may capture early, site-specific alterations that precede spirometry decline and are aligned with distinct long-term outcomes. Nevertheless, our findings are preliminary and validation in independent larger studies is required to confirm the indicated BAL-miRNAs as biomarkers in lung transplantation.

## 5. Conclusions

This study provides preliminary longitudinal evidence that BAL-derived miRNAs may serve as early biomarkers for CLAD development and subtype differentiation. Our findings support a role for BAL-derived miRNAs as early biomarkers of CLAD that can distinguish between BOS and RAS. These data provide an initial insight into the distinct biological mechanisms underlying chronic lung allograft dysfunction and lay the groundwork for future clinical intervention. The need for effective strategies to monitor and prevent the onset and progression of chronic lung rejection after lung transplantation is still a major shortcoming. In this scenario, miRNAs offer a dynamic, early and non-invasive opportunity to support current clinical protocols for the management of LT patients.

## Figures and Tables

**Table 1:** Clinical characteristics ^1^ of the LT patients’ series. Additional information is provided in Supplementary Table S1.

	Recipient Features			Donor Features		Surgery Features		Follow-Up @ 1 yr				Follow-Up @ 5 yr		Follow-Up @ 9 yr			
Patient ID	Disease	Age	LAS	DBD/DCD	Sex Mismatch	EVLP	W.I.	PGD_72 h	Best FEV1	AR	Status	CLAD	Status	CLAD	ReLT	Status	Follow-Up Time
LT_1	CF	26	32.0	DBD	N	N	Y	0	87	1	Alive	BOS	Alive	BOS		Alive	104
LT_2	ILD	64	31.3	DBD	N	N	Y	1	115	1	Dead	RAS	Dead	RAS		Dead	19
LT_3	CF	40	62.1	DBD	N	N	N	1	118	0	Alive	0	Alive	BOS		Dead	96
LT_4	CF	44	35.3	DBD	N	Y	Y	1	110	1	Dead	RAS	Dead	RAS		Dead	20
LT_5	CF	21	33.2	DBD	N	N	N	0	81	1	Alive	0	Alive	0		Alive	101
LT_6	CF	18	38.5	DBD	Y	N	N	2	94	0	Alive	0	Alive	BOS		Alive	96
LT_7	CF	33	33.5	DBD	Y	N	N	0	101	1	Alive	BOS	Alive	BOS	Y	Alive	100
LT_8	CF	32	35.5	DBD	N	N	N	0	108	0	Alive	0	Alive	0		Alive	103
LT_9	ILD	62	50.9	DBD	N	N	N	0	68	0	Alive	0	Alive	0		Alive	100
LT_10	ILD	43	68.6	DCD	Y	Y	N	3	55	1	Alive	BOS	Alive	BOS		Alive	98
LT_11	ILD	54	36.5	DBD	N	N	Y	3	74	0	Alive	0	Alive	0		Alive	98
LT_12	CF	24	43.2	DCD	Y	Y	N	3	86	1	Alive	0	Alive	0		Alive	98
LT_13	CF	37	33.3	DCD	Y	Y	N	1	97		Alive	RAS	Alive	RAS	Y	Alive	97
LT_14	CF	28	45.2	DBD	N	Y	N	2	74	0	Alive	0	Alive	0		Alive	97
LT_15	CF	21	38.7	DBD	N	N	N	1	80	0	Alive	0	Alive	BOS		Alive	97
LT_16	ILD	57	49.8	DBD	N	Y	Y	2	85	1	Alive	BOS	Dead	BOS		Dead	17

^1^ Abbreviations: yr, years; LT, lung transplant; CF, cystic fibrosis; LAS, lung allocation score; EVLP, extracorporeal lung perfusion; W.I., warm ischemia; PGD, primary graft dysfunction; DBD/DCD, donation after brain death/donation after circulatory death; FEV1, forced expiratory volume in 1 s; AR, acute rejection; Y/N, yes/no.

## Data Availability

The original contributions presented in this study are included in the article/Supplementary Materials. Further inquiries can be directed to the corresponding authors.

## Supplementary Materials

The following supporting information can be downloaded at: https://www.mdpi.com/article/10.3390/cells15070611/s1. Figure S1: Let-7e-5p and miR-30d-3p correlation with 5y-CLAD prognosis. Figure S2: Dynamics of microRNAs correlated with BOS development. Figure S3: The miR17-92 cluster member miR-19b-1-5p is an early biomarker of BOS risk for LT patients. Figure S4: Dynamics of microRNAs correlated with RAS development. Table S1: Additional patients details are provided. Table S2: K-means analysis. miRNAs belonging to each of the five k-means cluster are listed. Table S3: Predicted targets (from Supplementary Table S2) were imported into the STRING database, and an analysis was run against the whole human genome. Enriched processes were downloaded for Gene Ontology–Biological process, KEGG and Reactome. The top 15 terms for each category are listed here. Highlighted terms show the signaling presented in Figure 1d. Table S4: Univariate analysis (permutation test) was performed to explore the potential association of BAL-miRNAs to post-transplant-related clinical variable at the three time-points. Adjusted *p* values (adj-*p*-value) are from FDR correction. Table S5: The estimated marginal means analysis corrected for multiple comparison was used to search for BAL-miRNAs whose variation over time was significantly associated with BOS. C19MC miRNAs are highlighted in gray. Table S6: The estimated marginal means analysis corrected for multiple comparison was used to search for BAL-miRNAs whose variation over time was significantly associated with RAS. Table S7: Cox-regression analysis was performed to identify T0-, T1- or T2-BAL miRNAs associated with CLAD risk. *p* values were corrected for multiple comparison, applying the FDR method (*p*.adj).

## Abbreviations

The following abbreviations are used in this manuscript:

miRNA	microRNA
CLAD	Chronic lung allograft dysfunction
LT	lung transplantation
BOS	bronchiolitis obliterans syndrome
RAS	restrictive allograft syndrome
BAL	bronchoalveolar lavage
MP	machine perfusion
ECMO	extracorporeal membrane oxygenation
MV	mechanical ventilation
PGD	primary graft dysfunction
FEV1	forced expiratory volume in the first second
AR	acute rejection
RQ	relative quantities
PCA	principal component analysis
FDR	false discovery rate
ROC	receiver operating characteristics
AUC	Area under the curve
HR	Hazard ratio
HBEpC-c	human bronchial epithelial cells
CF	cystic fibrosis
ILD	interstitial lung disease
LAS	lung allocation score
DCD	donation after circulatory death
DBD	donation after brain death
EVLP	ex vivo lung perfusion
COPD	chronic obstructive pulmonary disease
BAL-EV	BAL derived extracellular vesicles
TLR	toll-like receptor
