# Supplementary material for: Early BAL microRNA Signatures Delineate Biological Trajectories Towards CLAD After Lung Transplantation
Source: Cells. 2026 Mar 30;15(7):611. doi: 10.3390/cells15070611 (PMC13072337; doi:10.3390/cells15070611)
Supplement: Supplementary file 1 [file cells-15-00611-s001.zip › R1_Supplementary Information.pdf]

Early BAL microRNA signatures delineate biological trajectories towards CLAD after lung transplantation

Gabriella Gaudioso, Sara Franzi, Riccardo Orlandi, Maria Rosaria De Filippo, Andrea Terrasi, Alessandra Maria Storaci, Nadia Mansour, Barbara Digiuni, Daniele Marchelli, Luca Valenti, Giorgia De Turris, Frederik von Herz, Giulia Garulli, Mario Nosotti, Letizia Corinna Morlacchi, Francesco Blasi, Alessandro Palleschi and Valentina Vaira

Supplementary Information

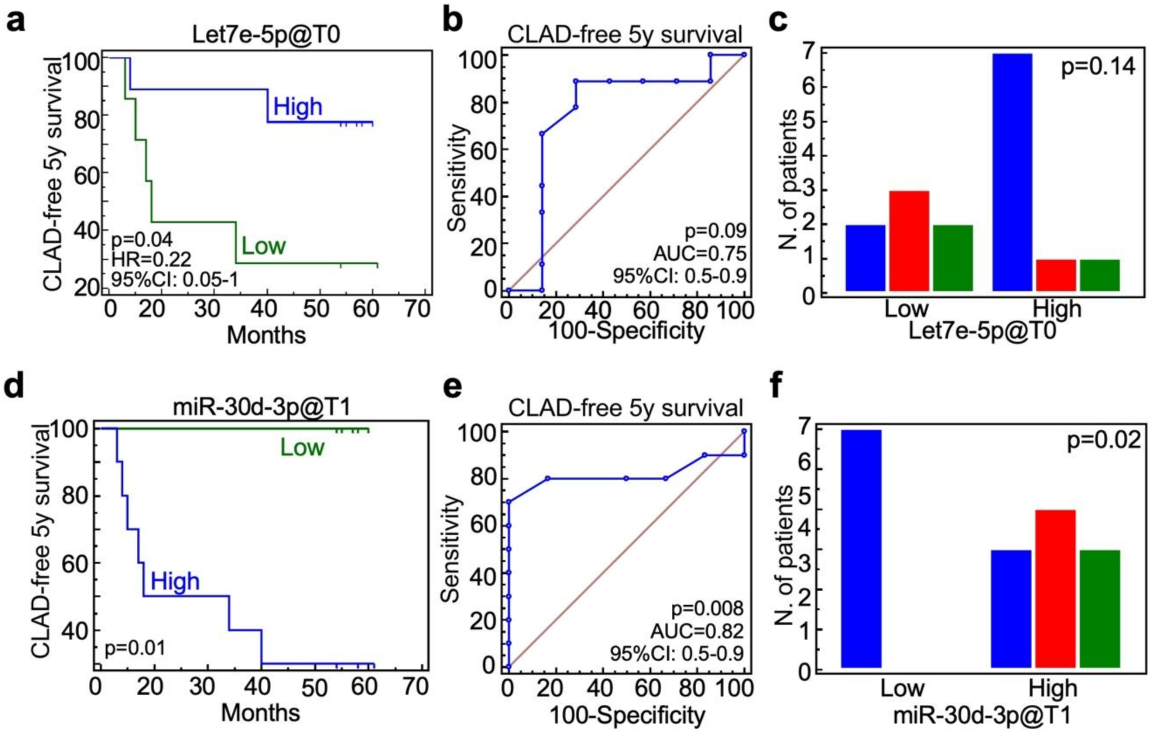

Suppl. Figure S1. Let-7e-5p and miR-30d-3p correlation with 5y-CLAD prognosis. (a) CLAD-free survival at 5 y is reduced in LT patients with low expression of let-7e-5p in BAL collected at T0P value is from log-rank test. (b) The diagnostic accuracy of let-7e-5p in discriminating LT patients according to CLAD outcome was analyzed using ROC curves. (c) LT patients were categorized as low or high let-7e-5p expressors (using the cut-off identified in Figure 3a) and the number of patients who remained stable or developed BOS or RAS is showed for each expression category. *P* value is from Chi-squared test. d) CLAD-free survival at 5 y is reduced in LT patients with high expression of miR-30d-3p in BAL collected at T1. P value is from log-rank test. (b) The diagnostic accuracy of miR-30d-3p in discriminating LT patients according to CLAD outcome was analyzed using ROC curves. (c) LT patients were categorized as low or high miR-30d-3p

expressors (using the cut-off identified in Figure 3f) and the number of patients who remained stable or developed BOS or RAS is showed for each expression category. *P* value is from Chi-squared test.

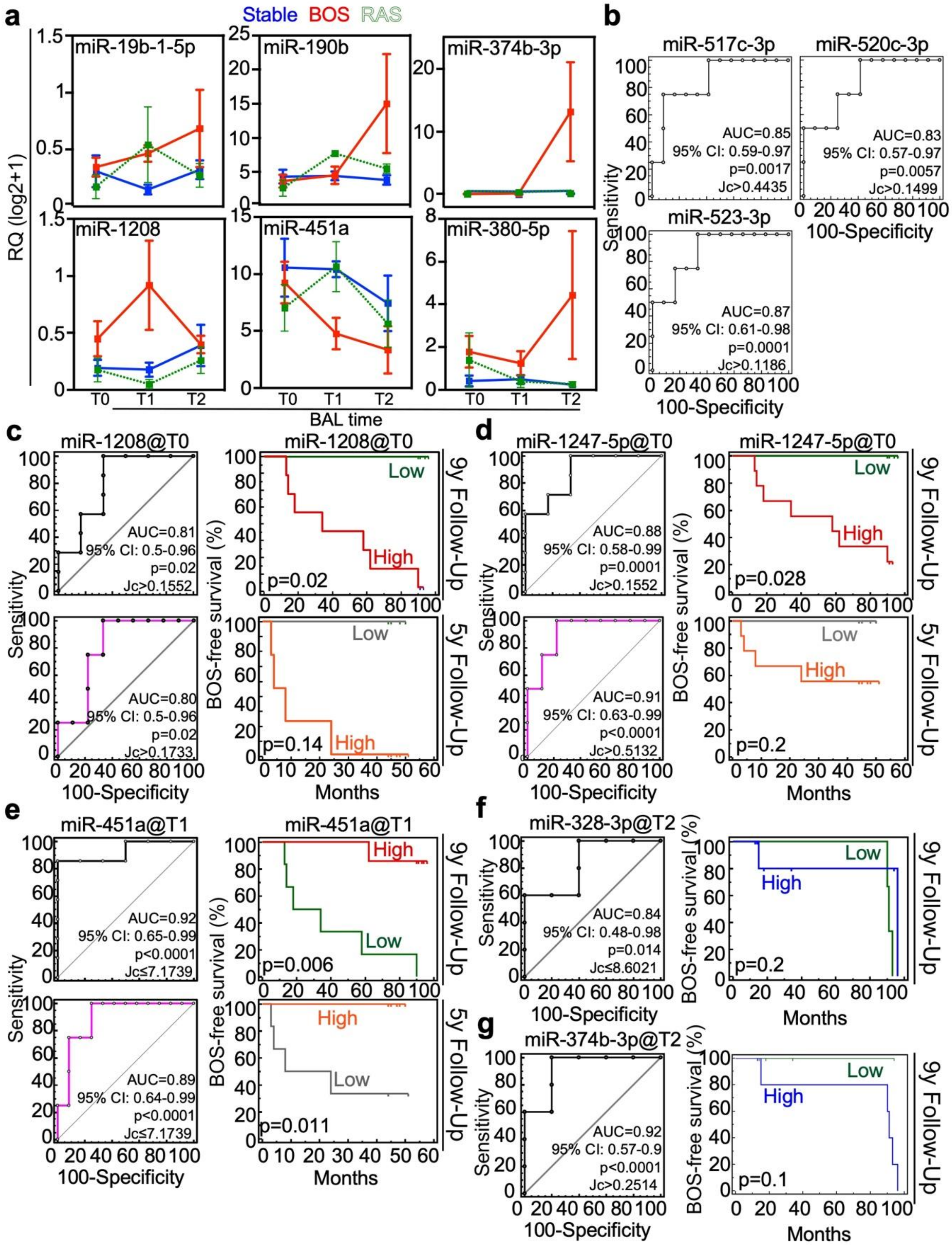

Suppl. Figure S2. **Dynamics of microRNAs correlated with BOS development.** (a) Differential expression of the indicated miRNAs in BOS respect with LT patients who remained stable or that developed RAS is shown. RQ, relative quantity. (b) C19MC miRNAs were tested as diagnostic biomarkers for BOS using ROC analysis and the Youden's criterion (Jc) was obtained. (c-g) ROC curves were generated to test the accuracy of the indicated miRNAs in classifying patients according to the development of BOS at 5y or at 9y from LT as indicated. The cutoff for miRNA expression was calculated using Youden's J statistic (Jc). Freedom from BOS (probability) was estimated using Kaplan-Meier curves in LT patients at 5y or at 9y from LT as indicated. Patients were sorted according to the ROC-generated cutoff into high- and low-miRNA groups. Indicated p values were computed by the log-rank test. AUC, area under the curve; CI, confidence interval; HR, hazard ratio.

a

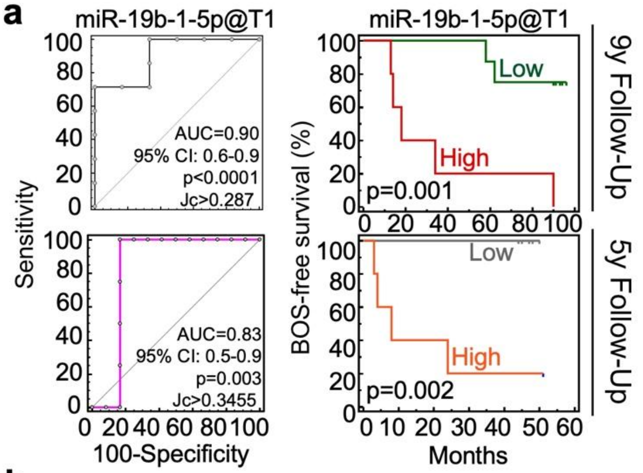

b

Clinical *w/* miR-19b1-5p model: overall fit p=0.047

Covariate	p	HR	95% CI of HR
AR in the first year	0.31	2.76	0.39 to 19.13
Disease other than CF	0.69	0.57	0.03 to 8.63
miR-19b-1-5p High	0.02	13.24	1.55 to 112.9
PGDmax: 2/3	0.74	1.52	0.12 to 18.25

Clinical model: overall fit p=0.56

Covariate	p	HR	95% CI of HR
AR in the first year	0.17	3.07	0.63 to 14.91
Disease other than CF	0.74	1.35	0.22 to 8.09
PGDmax: 2/3	0.66	0.70	0.14 to 3.45

c

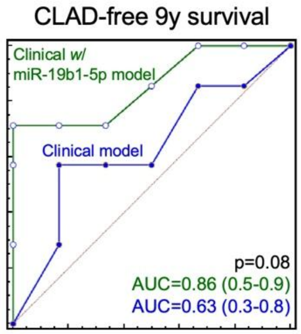

Suppl. Figure S3 The miR17-92 cluster member miR-19b-1-5p is an early biomarker of BOS risk for LT patients (a) ROC curves were generated to test the accuracy of the miR-19b-1-5p in classifying patients according to the development of BOS at 5y or at 9y from LT as indicated. The cutoff for miRNA expression was calculated using Youden's J statistic (Jc). Freedom from BOS (probability) was estimated using Kaplan-Meier curves in LT patients at 5y or at 9y from LT as indicated. Patients were sorted according to the ROC-generated cutoff into high- and low-miRNA groups. Indicated p values were computed by the log-rank test. (b,c) A multivariate Cox model was computed including (b, top panel) or not (b, lower panel) the miR-

19b-1-5p expression category. The accuracy of the two prediction models to identify LT patients who developed BOS, was then tested comparing the two ROC curves generated with the residual probabilities (risk scores computed by the Cox models) and the DeLong statistics. AUC, area under the curve; CI, confidence interval; HR, hazard ratio.

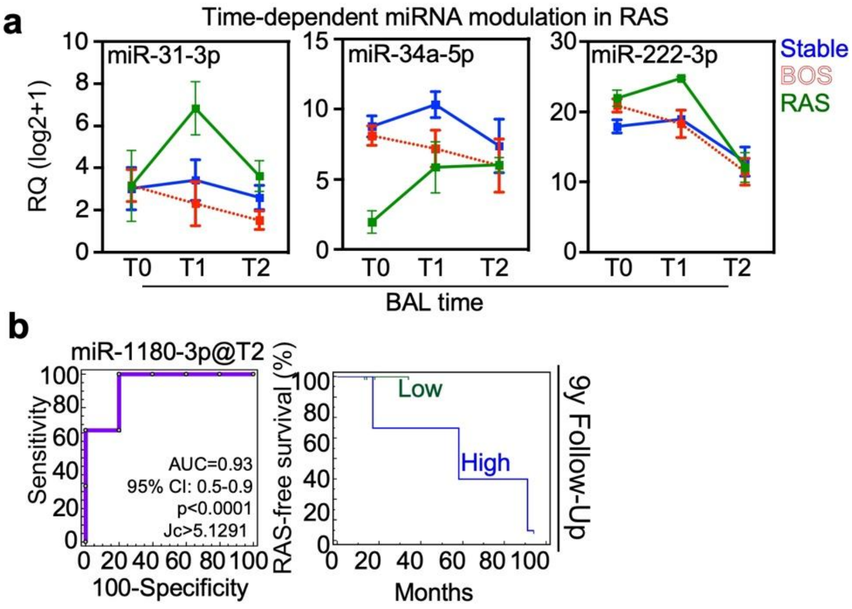

Suppl. Figure S4 Dynamics of microRNAs correlated with RAS development. (a) Differential expression of the indicated miRNAs in RAS respect with LT patients who remained stable or that developed BOS is shown. RQ, relative quantity. (b) The T2-upregulated miR-1180-3p was tested as diagnostic biomarkers for RAS using ROC analysis. The cutoff for miRNA expression was calculated using Youden's J statistic (Jc). Freedom from RAS (probability) was estimated using Kaplan-Meier curves in LT patients at 9y from LT as indicated. Patients were sorted according to the ROC-generated cutoff into high- and low-miRNA groups. AUC, area under the curve; CI, confidence interval.
